# Design, Preparation, and Evaluation of Osthol Poly-Butyl-Cyanoacrylate Nanoparticles with Improved In Vitro Anticancer Activity in Neuroblastoma Treatment

**DOI:** 10.3390/molecules27206908

**Published:** 2022-10-14

**Authors:** Liqing Zheng, Lixia Shen, Ze Li, Xiaoli Zhang, Miaomiao Wu, Yuanyuan Zhang, Jianhua Liu

**Affiliations:** 1 Department of Pharmacy, Hebei North University, Hebei Key Laboratory of Neuropharmacology, Zhangjiakou 075000, China; 2 Life Science Research Center, Hebei North University, Zhangjiakou 075000, China; 3 Department of Respiratory and Critical Care Medicine, The First Affiliated Hospital of Hebei North University, Zhangjiakou 075000, China

**Keywords:** osthol, SH-SY5Y, endocytosis, PBCA, nanoparticle, neuroblastoma

## Abstract

Osthol (osthole), known as a neuroprotective drug, has shown potent anticancer activity. However, the potential clinical application of osthol is limited due to its low water solubility and low bioavailability. Polybutyl cyanoacrylate (PBCA) has been widely used to improve the solubility of drugs with poor water solubility. In this study, an orthogonal experimental design (OED) was applied to design the preparation process of PBCA nanoparticles (NPs). Then, nanoparticles were prepared and evaluated in terms of physicochemical properties, in vitro release, and cellular uptake, etc. Further, the anti-cancer activity of osthol-PBCA NPs was demonstrated in SH-SY5Y cells. The pharmacokinetics and area under the curve (AUC) were investigated. The obtained osthol-NPs presented a spherical shape with a particle size of 110 ± 6.7 nm, a polydispersity index (PDI) of 0.126, and a zeta potential of −13 ± 0.32 mV. Compared with the free osthol, the drugs in osthol-NPs presented better stability and sustained release pattern activity. In vitro analysis using SH-SY5Y neuroblastoma cells showed that osthol-loaded nanoparticles displayed a significantly enhanced intracellular absorption process (three times) and cytotoxicity compared with free osthol (*p* < 0.05, increased 10–20%). The in vivo pharmacokinetic study revealed that the AUC of osthol-NPs was 3.3-fold higher than that of free osthol. In conclusion, osthol-PBCA NPs can enhance the bioactivity of osthol, being proposed as a novel, promising vehicle for drug delivery.

## 1. Introduction

Neuroblastoma (NB) is the most common pediatric solid tumor, originating from neural precursor cells. It most commonly arises in and around the adrenal glands. According to statistics, NB accounts for approximately 15% of malignant tumors in children and around 70% develop metastatic diseases [1]. Although surgical techniques, radiotherapy, and chemotherapy have made great progress, the long-term survival rate of NB is still less than 40%. Therefore, it is still necessary to explore effective NB treatments.

In the last two decades, natural product-based therapies have become popular for their effectiveness and potentially low toxicity. Osthol (osthole), 7-methoxy-8-(3-methyl-2-butenyl)-2H-1-benzopyran-2-one [2], is a natural coumarin extracted from ripe conidium fruits and has a wide range of pharmacological effects, such as anti-bacterial effects, vasorelaxant and neuroprotective properties, and antioxidant, anti-inflammatory, anti-osteoporosis, and anti-allergic effects [3,4], many of which have correlations with the primary or secondary prevention of cancer. Moreover, it also has anti-tumor activity in multiple types of cancer, such as breast cancer, endometrial cancer, ovarian cancer, gastric cancer, and lung cancer [5,6,7,8], with the mechanism of inhibition of the proliferation and induction of apoptosis and cell cycle slowdown; apart from this, osthol has a synergistic effect with other anti-tumor substances, such as FOLFOX, cisplatin, or DOX [9]. Interestingly, it has been reported that osthol has a direct killing effect on neuroblastoma (NB) cells by blocking voltage-gated Na^+^ channels in mouse neuroblastoma N2A cells and inhibiting the L-type calcium current in a mouse neuroblastoma and rat glioma hybrid cell line, NG108-15 [10,11]. However, low bioavailability due to instability, poor absorption and poor water solubility, irritation in the gastrointestinal tract, and a short half-life have limited osthol’s therapeutic applications [12,13,14]. In recent years, osthol preparation methods have been extensively studied; methods such as injections, inclusion compounds, solid dispersants, ointments, gel agents, patches, and other dosage forms have been developed to improve the solubility, stability, and bioavailability of osthol. However, these unfavorable properties of osthol remain unresolved. Recently, the solubility and bioavailability of osthol was correspondingly enhanced due to the formation of nanoemulsions to enhance the treatment of Alzheimer’s Disease via intranasal administration [14]. This study suggests that nanotechnology is a potential approach to enhance the solubility and bioavailability of traditional Chinese medicine (TMC), becoming a research hotspot. 

Efficient encapsulation of the drugs not only allows us to improve the water solubility, improve their efficacy, and reduce their side effects, but also to achieve targeted or directional drug delivery according to the different particle size distributions in vivo [15,16,17]. Polybutyl cyanoacrylate (PBCA) is a polymer of α-cyanoacrylate (α-BCA), which is a common representative of the PACA family, first used in 1979. PBCA has been used as a surgical adhesive in clinical practice for its fast polymerization mechanism and biodegradability [15]. Studies have shown that PBCA nanoparticles (NPs) are ideal carriers for the delivery of drugs due to their biocompatibility, organ targeting, lack of immunogenicity, low toxicity, and stability, and can achieve targeted drug delivery via easy chemical or physical modification [18]. Meanwhile, PBCA NPs can achieve controlled release for their appropriate chain lengths, and maintain better bioactivity for the drugs loaded [19]. Additionally, PBCA nanoparticles are easy to prepare and purify [16]. It has been reported that particles between 40 and 200 nm in size may be absorbed through paracellular absorption or endocytosis mechanisms [18]. The particles could overcome multi-drug resistance [20]. Because of these advantages, PBCA has become a candidate carrier material for drug delivery systems.

The orthogonal experimental design, introduced by Taguchi G, is a design method that uses an orthogonal table to screen multiple factors with multiple levels [21]. Finally, through the analysis of variance and range analysis, we can determine the primary and secondary relationships of each factor. The main purpose is to seek the optimal level [22]. An orthogonal experimental design is a conventional method for experimental design, which was applied in this study to optimize the preparation conditions of osthol-loaded PBCA-NPs.

In this study, osthol was efficiently encapsulated with PBCA to improve the bioavailability and water solubility. Moreover, the physicochemical properties, anti-cancer activity in SH-SY5Y cells, and AUC were investigated. The aim of this study was to optimize the preparation conditions of osthol-PBCA-NPs and to assess the anti-cancer activity in NB cancer cells. The study will provide a theoretical basis for the further exploration and utilization of osthol in the aspect of NB treatment.

## 2. Materials and Methods

### 2.1. Materials

Osthol (high performance piquid chromatography (HPLC) purity > 95%) was purchased from Qingze Lvsheng Plant Co., Ltd. (Nanjing, China). Poloxamer-127 and Trypsin were obtained from Sigma (Schnelldorf, Germany) and the poly-butyl cyanoacrylate was purchased from Shunkang Medical Adhesion Co., Ltd. (Beijing, China). DMEM-F12 medium was purchased from Hyclone (Logan, UT, USA), Fetal Bovine Serum (FBS) was obtained from Gibco (Grand Island, NY, USA), and 3-(4, 5-Dimethylthiazole-2)-2, 5-diphenyltetrazolium bromide (MTT) was purchased from Amresco Inc. (Solon, OH, USA). Streptomycin Mixture (100×), sucrose, glucose, and RIPA protein lysate were purchased from Beijing Solaibo Technology Co., Ltd. (Beijing, China). Sodium azide and dimethyl sulfoxide were obtained from Shanghai Sangong (Shanghai, China) and used as purchased. Human SH-SY5Y cell neuroblastoma was purchased from the Cell Center of the Peking Union Medical College (Beijing, China).

### 2.2. Osthol Analysis

#### 2.2.1. Determination of the Optimum Detection Wavelength for Osthol Analysis

Osthol in a methanol solution was scanned over the wavelength range of 200–400 nm using a UV spectrophotometer (Tecan safire^2^, Männedorf, Switzerland) to determine the wavelength of maximum adsorption for osthol.

#### 2.2.2. Chromatographic Conditions and Calibration Curve

Osthol concentrations were determined on an Agilent 1100 HPLC (Santa Clara, CA, USA) equipped with an Agilent non-polar YWG-C18 column (4.6 mm × 250 mm, 10 μm, Santa Clara, CA, USA). The mobile phase was methanol–water (80: 20 *v*/*v*; isocratic flow). The flow rate, detection wavelength, and injection volume were 1 mL·min^−1^, 322 nm, and 10 μL, respectively. The column temperature was kept constant at 20 °C and the elution time of osthol in HPLC was 8 min.

Osthol was dissolved in methanol to obtain 25 μg·mL^−1^ as a stock solution and diluted to varying concentrations of 0.15, 0.31, 0.625, 1.25, 2.5, 5, and 10 μg·mL^−1^ using the standard addition method. The UV absorption peak area (Y) of each concentration (X) was obtained by using a UV HPLC detector at a wavelength of 322 nm. The calibration curve was established by linear regression.

### 2.3. Preparation of Osthol-PBCA NPs

According to the polymerization principle of the butyl cyanoacrylate monomers, the interfacial polymerization method was employed. Osthol powder was dissolved in acetone and dispersed for 15 min to obtain 5 mL of a 1 mg·mL^−1^ solution; α-BCA dissolved in trace ethyl acetate was added as the organic phase. This organic phase was slowly dropped to an aqueous phase using an electronic micropump, WLB-78-A (Zhejiang, China) (at a rate of 0.25 mL·min^−1^, within 20 min), at a constant temperature of 19 °C. The aqueous phase consisted of deionized water containing the surfactant Poloxamer-127 to maintain a stable emulsion. The pH was initially adjusted to 2.5 with a 0.1 mol·L^−1^ dilute hydrochloric acid solution, and the solution was stirred continuously at 19 °C. After stirring for 1 h, the solution pH was adjusted to 7.2 using a 0.1 mol·L^−1^ NaOH aqueous solution and stirred again for 1 h. A milky microemulsion was obtained under the high-speed stirring shear force for 2 h in a sealed container, also at 19 °C. The solvent was removed in a rotary evaporator (5 min at 30 °C under vacuum), leaving the osthol-PBCA NP mixture with a light blue and slightly opaque color. The osthol-free PBCA NPs were prepared by a similar method without osthol being added during the whole preparation process. After filtration with a 0.45 μm pinhole filter, lyophilization of the solution was carried out without adding any lyophilization protectant. The resulting osthol-PBCA NPs were stored at 4 °C until further use. 

#### 2.3.1. Single-Factor Experiment

In the single-factor experiment, particle size and dispersion were examined to determine the best preparation parameters: stirring rate, temperature, and the pH value of the solution (results not shown). One surprising observation was that the stirring rate was the main factor in controlling the NP sizes. To study the effect of the stirring rate on particle size, the NPs were prepared with stirring rates of 800 r·min^−1^, 1000 r·min^−1^, and 1200 r·min^−1^, respectively, while keeping the other parameters constant. The particle size was measured with a Malvern particle size analyzer (Nano-ZS, Malvern Instruments Ltd., Malvern, UK). 

#### 2.3.2. Optimization of the Orthogonal Design

Based on the results from the single-factor pre-experiment, we selected the most suitable level of each factor (Table 1). When osthol-PBCA NPs were prepared via interfacial polymerization, we found that the stirring speed (A), aqueous phase pH (B), the ratio of osthol (5 mg): α-BCA (C), and the dose of Poloxamer-127 (D) were important process parameters that affected the encapsulation efficiency and dispersibility in the preparation process. The orthogonal experimental design table L^9^ (34) was used to optimize the preparation method [23], with the encapsulation rate as the index. The orthogonal design assistant V 3.0 software was used to identify the best experimental parameters. 

### 2.4. Evaluation of Nanoparticles 

#### 2.4.1. Particle Size and Zeta Potential

PBCA-NPs were prepared according to the optimized conditions mentioned above. First, 20 μL osthol-PBCA NPs were added to 1 mL of phosphate-buffered saline (PBS) in triplicate. The particle size, zeta potential, and polydispersity index (PDI) of the PBCA NPs were measured using a Malvern Zeta Size Nano-ZS set at 620 nm (Nano-ZS, Malvern Instruments Ltd., Malvern, UK). The data are shown as mean ± standard deviation (SD) (*n* = 3).

#### 2.4.2. Encapsulation Efficiency (EE) and Drug Loading (DL) 

Drug encapsulation and loading efficiencies were determined by HPLC (Agilent 1100, Agilent Technologies Inc., Santa Clara, CA, USA), and using the osthol standard solution. The freshly prepared aqueous suspension was diluted to 10 mL with purified water in a 10 mL volumetric flask to obtain the test solution, and then 1 mL osthol-PBCA NP suspension was taken out and separated by ultra-centrifugation (15,000 r·min^−1^, 4 °C, 60 min), and the concentration of the remaining free osthol in the supernatant was determined by HPLC (Agilent 1100, Agilent Technologies Inc., Santa Clara, CA, USA) to calculate EE (%). For the DL test, the sediments above were freeze-dried, weighted, and then destroyed and extracted by methanol. NPs were added to 1 mL methanol and ultrasonicated in an ultrasonic cleaner (KS-600, China) for 15 min; no complete NPs were detected by transmission electron microscopy (TEM). After a short period of centrifugation (15,000 r·min^−1^, 4 °C, 5 min), the concentration of osthol in the methanol supernatant was determined by HPLC (Agilent 1100, Santa Clara, CA, USA). The EE and DL were determined using Equations (1) and (2), respectively [15].

(1) $$EE\%=\frac{Total\;drug\;amoumt-unloaded\;drug\;amoumt}{Total\;osthol\;amoumt}\times 100\%$$

(2) $$DL\%=\frac{Drug\;loaded\;in\;NPs}{Total\;drug\;NPs}\times 100\%$$

#### 2.4.3. TEM Studies of the Osthol-PBCA NPs

The osthol-PBCA NPs were dispersed by shaking in water at a concentration of 5 mg· mL^−1^. The sample was subsequently dropped onto a 200-mesh carbon plating film copper grid and excess water was removed using filter paper. After air drying, the sample was stained with 1% phosphotungstic acid. The morphology of the sample was obtained using a TEM at 80 kV (H-7650 HITACHI, Hitachi High-Tech Co., Tokyo, Japan). 

#### 2.4.4. Differential Scanning Calorimetry (DSC) Analysis

DSC curves of osthol, blank-PBCA NPs, osthol-PBCA NPs, and a physical mixture were measured with a DSC-204 instrument (Netzsch, Germany). The heat flow scans were recorded from a temperature of 40 °C to 200 °C, at a heating rate of 10 °C/min, in an atmosphere of nitrogen. 

#### 2.4.5. Fourier Transform Infrared (FTIR) Analysis

The powder-like samples were mixed with KBr and prepared into pellets. The FTIR spectra of osthol, blank-PBCA NPs, osthol-PBCA NPs, and a physical mixture were measured with a Bruker-MPA FTIR system (Bruker, Switzerland), in the region from 4000 cm^−1^ to 400 cm^−1^.

#### 2.4.6. Influencing Factors Test

The influencing factors tests were prepared according to the guidelines for stability testing of the Chinese Pharmacopeia (2020) [24]. Osthol-loaded PBCA suspensions can be prepared as a freeze-dried powder in order to enhance their stability and facilitate storage and transportation. The stability of osthol, osthol-PBCA NP suspensions, and the freeze-dried powder were studied at the same time. Osthol-PBCA NP lyophilized powder was dispersed in water as osthol-PBCA NP suspensions at a concentration of 1 mL of the suspension containing 1 mg of osthol. Ten equal samples of osthol (1 mg) and osthol-PBCA NP lyophilized powder suspensions (1 mL) were taken and treated for 0, 2, 7, 14, and 30 days, respectively, and then analyzed by HPLC. The influencing factors on the stability of the osthol-NP suspensions and the powdered material were investigated at a light intensity of 3000 lux [25] in a light incubator (HPG-280 B, China) (optical stability test) and at 60 °C [24,25] over a period of 30 days in a Drug Stability Tester (WD-B, China) (*n* = 10). The particle size, zeta potential, and PDI of osthol nanoparticle suspensions were measured with a Malvern particle size analyzer (Nano-ZS, Malvern Instruments Ltd., Malvern, UK). 

### 2.5. In Vitro Release Study 

Due to the poor solubility in water of osthol and good solubility in methanol, osthol was dissolved in methanol to observe the release process better in vitro. The release of osthol from osthol-PBCA NPs was studied by the dynamic dialysis technique in vitro. First, 5 mL of the osthol in methanol solution and the osthol-PBCA NP suspension, which contained the same amount of osthol (1 mg·mL^−1^), were placed into dialysis bags (8000–14000 MW cut-off). The pH in extracellular and normal tissues ranges from 7.2 to 7.4, and that in malignant tumor tissues ranges from 6.5 to 6.9. Acidification was prevalent in the tumor extracellular microenvironment. The bags were then immersed separately in 200 mL of PBS (pH 7.4 and pH 6.9) at a stirring rate of 100 r·min^−1^ and at 37 ± 0:5 °C. An aliquot of 0.5 mL of the dissolution medium was withdrawn at time intervals of 0.25, 0.5, 1, 2, 4, 6, 8, 10, 12, 24, 36, 48, 60, and 72 h. After each sampling, the dissolution medium was replenished with an equal volume of fresh PBS. All the dissolution samples were filtered through a 0.22 μm Millipore filter before HPLC analysis. The drug cumulative release rate (CRR) was calculated by using Equation (3), and the drug release–time curve was obtained based on the CRR.

(3) $$CRR\left(\%\right)=\frac{W_{release}}{W_{total}}\times 100\%$$

where *W_total_* (mg) and *W_release_* (mg) are the quantities of the drug loaded into the nanoparticles and the drug released from the nanoparticles into the medium at different time intervals, respectively.

### 2.6. Cell Absorption Studies In Vitro 

The SH-SY5Y cellular uptake of osthol solutions and osthol-PBCA NPs was determined quantitatively. Osthol was dissolved in a serum-free medium to obtain a 10 μg·mL^−1^ solution, and the solution was filtered to remove bacteria. SH-SY5Y cells were seeded in 96-well culture plates with cell densities of 1 × 10^5^ cells/well. After the cells reached 80% confluence, the medium was replaced with a fresh serum-free medium containing the osthol solution or osthol-PBCA NPs at various periods, ranging from 0.5 to 16 h at 37 °C and with 5% CO_2_ in the incubator. After they were incubated with 100 μL of osthol serum-free medium solution or osthol-PBCA NPs containing 10 μg·mL^−1^ osthol for 0.5, 1, 2, 3, 4, 5, 6, 7, 8, 12, and 16 h, the cells were washed three times with cold PBS buffer, and then lysed by adding RIPA protein lysate. The cells were kept on ice for 30 min while shaking 2–3 times.

In order to study the uptake of drugs into cells, 4 mL of CHCl_3_ was added to 100 μL lysate described earlier to extract osthol from cell lysate. The organic part was separated and dried under N_2_; then, we re-dissolved the residue in the HPLC mobile phase and examined it by HPLC. We used first-order reaction kinetics to calculate the rate constant.

### 2.7. Exploring the Cell Endocytosis Mechanism of NPs

Endocytosis inhibitors were used to study the endocytosis mechanism of NPs. Chlorpromazine inhibited clathrin-mediated endocytosis, sucrose inhibited fluid-phase endocytosis, and NaN_3_ inhibited energy metabolism. SH-SY5Y cells were pre-incubated with endocytic inhibitors at concentrations of 7 μg·mL^−1^ of chlorpromazine, 0.01 mol·L^−1^ of NaN_3_, and 0.45 mol·mL^−1^ of sucrose, without causing toxicity to the cells [26,27]. Following a pre-treatment time of 30 min, the cells were cultured with the newly prepared solutions of either 10 μg·mL^−1^ osthol or osthol-PBCA NPs for 4 h at 37 °C. Meanwhile, the cells were cultured with osthol or osthol-PBCA NPs at 0 °C in low temperature test. Subsequently, the cells were washed with PBS buffer three times and treated as described above. Cells containing NPs without inhibitors were used as controls. 

### 2.8. Determining Cytotoxicity Effects In Vitro

To evaluate the cytotoxicity of blank PBCA-NPs, osthol, and osthol-loaded PBCA-NPs, the MTT test was carried out. For this purpose, SH-SY5Y cells were cultured in DMEM-F12 medium supplemented with 10% FBS and 1% penicillin/streptomycin antibiotics. Logarithmic growth phase cells were seeded into 96-well plates, with 1 × 10^5^ cells per well, and incubated at 37 °C under 5% CO_2_. After 24 h incubation, the cells were treated with different concentrations of osthol (1.25, 2.55, 10, 20 μg·mL^−1^, *n* = 4) or osthol-loaded PBCA suspensions, respectively. After 24 h and 72 h, the cell survival rates were determined by the MTT assay. Cells without any treatment were used as controls. 

After incubation, 20 µL of 5 mg·mL^−1^ MTT was added to the 96-well plates, and the cells were incubated (5% CO_2_, 37 °C) for another 4 h. The MTT solutions were carefully removed and 180 µL of dimethyl sulfoxide (DMSO) was added to dissolve the formazan crystals. The mixture was shaken for 10 min. The absorbance was measured at 570 nm using a UV spectrophotometer (Tecan safire^2^, Männedorf, Switzerland). The cell viability was calculated using Equation (4):

(4) $$Cell\;survival\;rate\;\left(\%\right)=\frac{Atest\;}{Acontrol}\times 100\%$$

where *A_test_* and *A_control_* are the absorption of the cells in the test group and the absorption of the cells in the control group at 570 nm, respectively.

### 2.9. Assessment of Membrane Integrity

Lactate dehydrogenase (LDH) is a soluble cytoplasmic enzyme that is released into the extracellular space when the plasma membrane is damaged [28]. The leakage of LDH is considered to be a significant cell death marker. The membrane integrity of SH-SY5Y cells was evaluated using an LDH cytotoxicity detection kit. Briefly, SH-SY5Y cells were seeded into a 96-well plate with 1 × 10^5^ cells per well and then treated with 10 μg·mL^−1^ osthol and/or 10 μg·mL^−1^ osthol-NPs for 24 h. Untreated cells were used as controls (*n* = 4). Subsequently, 100 μL of supernatant from each well was transferred into another 96-well plate in triplicate, and we added 100 μL LDH reaction mixture. After incubation at 37 °C under 5% CO_2_ for 3 h, the optical density of each well was determined at a wavelength of 490 nm using a UV spectrophotometer (Tecan safire^2^, Männedorf, Switzerland).

### 2.10. Pharmacokinetic (PK) and Aera under the Curve (AUC) study in Rats

Sprague Dawley (SD) rats (150 ± 5 g) were purchased from Hebei North University and fed via standard procedures. Eighteen mice were randomly divided into two groups. They were administered a single dose of osthol (5 mg·kg^−1^) or osthol-PBCA NPs containing 5 mg/kg osthol via tail vein, respectively. A 0.5 mL blood sample was collected through the inner canthus at 0.125, 0.25, 0.5, 1, 2, 4, 6, 8, and 12 h after administration. Then, blood samples were centrifuged at 3000 r·min^−1^ for 10 min to separate plasma. Then, 100 μL of plasma was transferred to a 1.5 mL centrifuge tube, followed by adding 200 μL acetonitrile. After being vortexed for 5 min, the samples were centrifuged at 10,000 r·min^−1^ for 10 min at 4 °C. The supernatant was collected and determined by HPLC. The plasma drug concentration–time curve was drawn using Origin Pro 8.1 and PK parameters were calculated by the software 3P87. 

### 2.11. Statistical Analysis

SPSS 20.0 software and one-way ANOVA were used to analyze the data. All data are represented as the mean ± standard deviation (SD, *n* = 4). Differences between groups were considered significant at the *p* < 0.05 level.

## 3. Results and Discussion

### 3.1. Osthol Determination Method

**Figure 1:**
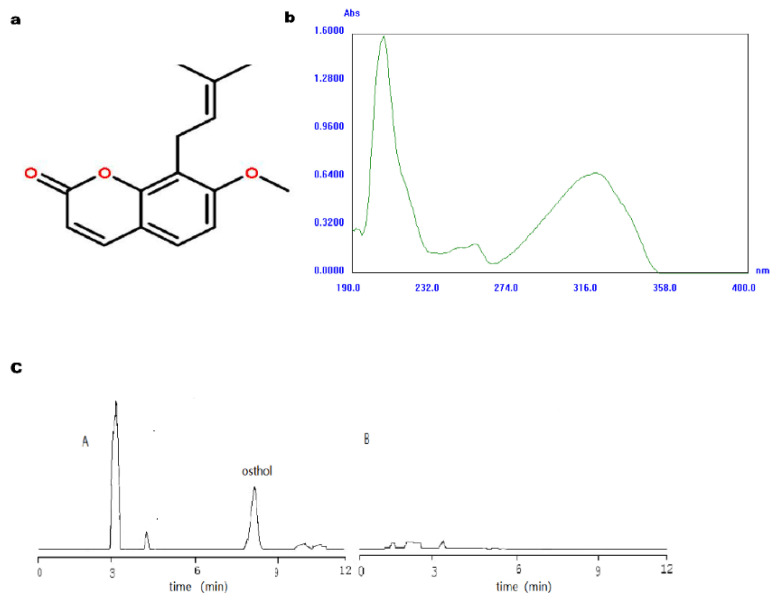
Structure, UV–Vis absorption spectrum, and HPLC chromatogram of osthol. (**a**) is chemical structure of osthol; (**b**) is the Uv absorption wavelength scan of osthol; (**c**) is the chromatogram of osthol sample (A) negative control (B).

The osthol structure is shown in Figure 1a. Osthol has its maximum absorbance at 322 nm. Other excipients have no obvious absorption at this wavelength and do not cause any interferences with the absorbance value measured at this wavelength (Figure 1b). The HPLC chromatogram of osthol is shown in Figure 1c. In Figure 1c, A is the chromatogram of osthol, and B is the chromatogram of a negative control. The elution time of osthol is 8 min. The linear regression equation Y = 106X + 812,313 (r = 0.9997) was obtained for the osthol peak area (Y), which allowed us to obtain the corresponding concentrations (X) for osthol. A good linear relationship in the concentration range of 0.39~25 μg·mL^−1^ was observed, as well as the reasonable recovery and precision of the method (RSD = 0.90%).

### 3.2. Preparation of Osthol-PBCA NPs

#### 3.2.1. The Relationship between Stirring Rate and Particle Size

**Figure 2:**
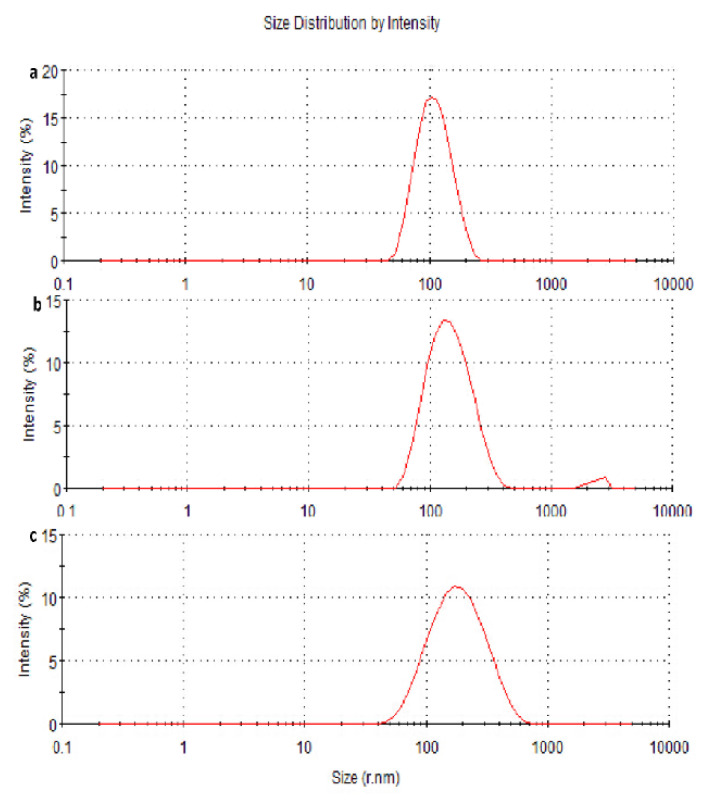
Single-factor investigation: influence of rotational speed on particle size. The size distributions of nanoparticles (**a**–**c**) were assessed at stir rates of 800, 1000, and 1200 r·min^−1^, respectively.

The NPs were prepared at stirring rates of 800, 1000, and 1200 r·min^−1^. The average particle size was 163.4, 137.3, and 101.8 nm, with a particle size distribution (PSD) of 102, 62, and 35 nm, respectively (Figure 2, Table 1). The particle size and PSD decreased with increasing stirring rate. 

#### 3.2.2. Optimization of the Preparation Process by Orthogonal Design

The four-factor three-level results are shown in Table 2. An L^9^ (34) orthogonal experimental design was used to optimize the osthol-PBCA NPs’ preparation. Encapsulation rates were used as the index, as shown in Table 3. The analysis of variables A−D is shown in Table 4. After analysis, the R value was used to determine the primary and secondary influencing variables: A > C > D > B. Variable A: K3 > K2 > K1, variable B: K2 > K3 > K1, variable C: K2 > K3 > K1, and variable D: K3 > K2 > K1. This indicates that the best design was A3B2C2D3. In conclusion, stirring speed had a significant effect on the drug encapsulation rate (*p* < 0.05), while the other three variables had no significant effect (*p* > 0.05). Therefore, the optimal conditions for osthol-loaded PBCA nanoparticles’ preparation were as follows: osthol dosage of 5 mg, a stirring rate of 1200 r·min^−1^, solution pH of 4, α-BCA dosage of 15 μL, dosage of Poloxamer-127 of 40 mg, an ethyl acetate dosage of 200 μL, and a reaction time of 2 h. According to the analysis of variance of the orthogonal experimental data, stirring speed has a significant effect (*p* < 0.05) on the EE%. The reason may be that the different mixing speeds have different shear forces, and shear forces have a significant impact on the sizes of interfacial polymerized products.

### 3.3. Characterizations of Osthol-PBCA NPs

Osthol-PBCA NPs were prepared according to the optimum preparation method. Because BCA could be physically encapsulated with many different types of drugs, BCA has been widely used in nano drug delivery systems found among various types of poly-alkyl-cyanoacrylate nanoparticles, including polymethyl, polyethyl, polypropyl, polybutyl, etc. [20]. Many studies have demonstrated that PBCA NPs are excellent drug carriers; they also enhance therapeutic effects, reduce side effects, and overcome multi-drug resistance to chemotherapeutics in cancer therapy [16,29]. The suspension with 5% osthol was transferred to a penicillin bottle and pre-frozen at −80 °C for 24 h, and then subjected to vacuum freeze drying (vacuum below 0.1 MPa, temperature at −50 °C, and a drying time of 10 h). A white, freeze-dried powder of the osthol-loaded PBCA NPs was recovered and sealed for further use.

#### 3.3.1. Particle Size Measurements and Morphology of the Nanoparticles

**Figure 3:**
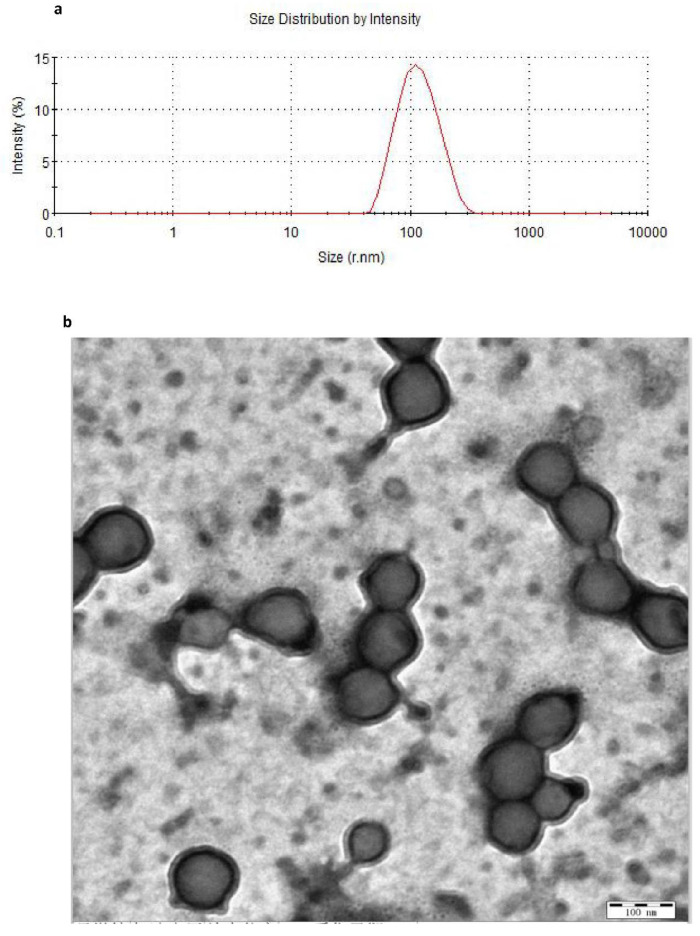

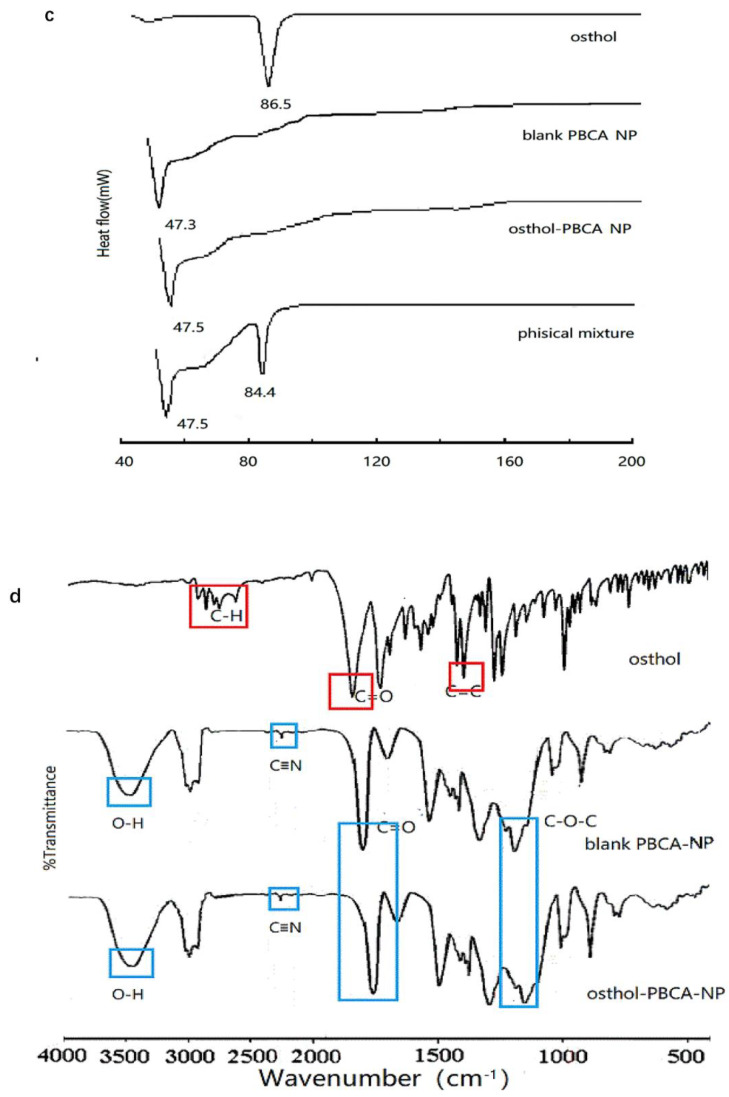
Particle size and morphology of the nanoparticles. (**a**) Osthol-PBCA NPs prepared were 110 nm ± 6.7 nm after screening by an orthogonal design study. (**b**) Morphology of the nanoparticles obtained by TEM (×30,000). (**c**) DSC and (**d**) FTIR spectrum of osthol, PBCA, and osthol-PBCA NPs.

In this study, the medium changed from colorless to milky white, which indicated the completion of the polymerization of BCA monomers [30]. The drug-loaded NPs were characterized using TEM and dynamic light scattering techniques. Figure 3 shows that osthol-PBCA NPs had a near-spheroid particle shape, with a particle size of around 100 nm, smaller than the size of 110 nm determined by light scattering techniques (Table 5). A difference of 10% is not unusual when using two techniques that are based on completely different measurement phenomena.

The size is a critical factor for intra-tumoral penetration and in vivo pharmacokinetics, including cellular uptake, biodistribution, and the circulation half-life of nanoparticles [31]. Nanoparticles smaller than 300 nm can effectively enter the target cells to achieve pharmacological functions [32]. The average sizes of the osthol-PBCA NPs prepared were 110 ± 6.7 nm (*n* = 3), and the range was narrow; thus, they could effectively pass through the cell membrane and enter the target cells.

#### 3.3.2. Zeta Potential and PDI Measurements

The zeta potentials and PDI of osthol-PBCA NPs and blank PBCA NPs are shown in Table 5. The zeta potentials of blank PBCA NPs and osthol-loaded PBCA NPs were −7 ± 0.40 mV and −13 ± 0.32 mV, respectively (*n* = 3). Osthol-PBCA NPs’ zeta potentials were more negative compared to the drug-free PBCA NPs. This is related to the negative charge of osthol encapsulated in the nanoparticles. The PDIs of blank PBCA-NPs and osthol-loaded PBCA NPs were 0.123 and 0.126, respectively (*n* = 3). The NPs were well dispersed. This suggests that PBCA is a promising nanodrug carrier.

#### 3.3.3. Encapsulation Efficiency (EE) and Drug Loading Efficiency (DL)

Following optimum preparation technology guidelines, three batches of osthol-NPs were prepared in parallel. The EE and DL of the three batches are shown in Table 5. The mean EE and DL were 80.59% and 40%, respectively. Despite the low water solubility of osthol [33], 40% DL can still be achieved. Therefore, improving the DL efficiency is critical for achieving a therapeutic effect. This confirms that the surface polymerization method can effectively be used to prepare osthol-loaded PBCA NPs. A low DL leads to a poor therapeutic effect and poor drug release curves, which makes drug loading efficiency a key factor in whether the polymer can be used as a drug carrier [34]. This method is simple and feasible, and provides a new idea for the realization of nano-traditional Chinese medicine.

#### 3.3.4. DSC and FTIR Analysis

The DSC of osthol crystalline (Figure 3c) showed a single peak of a melting endotherm at 86.5 °C. The physical mixture of osthol and PBCA showed an endotherm at 84.4 °C, with a smaller peak, whereas the osthol-NPs had no distinct melting endotherm. DSC analysis implied that it was not a simple physical mixture of osthol and PBCA, but molecular encapsulation of osthol within the PBCA-NPs.

FTIR spectra of osthol, PBCA-NP, osthol-PBCA NP, and physical mixtures are shown in Figure 3d. The IR spectra showed characteristic peaks of osthol C=O stretching near 1700 cm ^−1^, C−C stretching near 1600 cm ^−1^ and 1500 cm^−1^, and aromatic H stretching and aliphatic C−H stretching near 3000 cm^−1^. The absorbance bands of blank PBCA-NPs were at approximately 2200 cm^−1^, 1750 cm^−1^, and 1100 cm^−1^ and were assigned to the stretching vibrations of CN, C=O, and C−O−C, respectively. The peak at 3300 to 3500 cm^−^^1^ was the O−H stretching of the hydroxyl group. FTIR analysis confirmed that osthol had been loaded successfully into the PBCA-NPs, and did not simply absorb at the NP surface.

##### Influencing Factors Test

**Figure 4:**
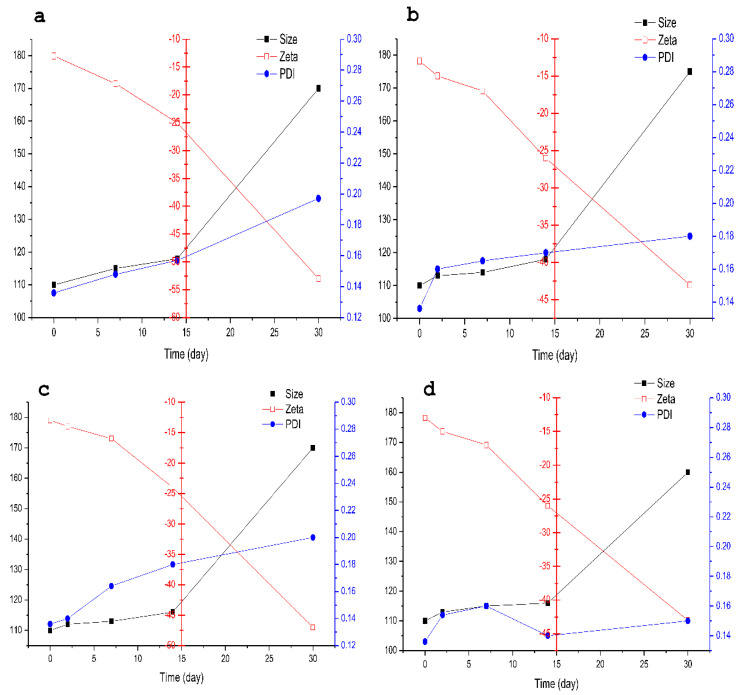
Particle size, zeta potential, and PDI of osthol nanoparticle suspensions and powder suspensions. Suspensions (**a**) at light intensity of 3000 lx for 30 days and (**b**) at 60 °C for 30 days. Power (**c**) at light intensity of 3000 lx for 30 days and (**d**) at 60 °C for 30 days.

The osthol content in the NPs was extracted by methanol and determined by HPLC. The results are presented in Figure 4 and Table 6. The intense light test showed that the osthol content in osthol-NP suspensions and powdered samples was 86% and 94%, compared with 32% for the osthol solution after 30 days of irradiation. The high-temperature test indicated that the osthol content in osthol-NP suspensions and powdered samples was 84% and 90%, compared to 28% for the osthol solution, confirming that the influence of high optical and thermal energy was prevented by the osthol loaded during nano crystallization. The size of NPs increased, and zeta potential decreased with time; they changed significantly after 14 days. The influencing factors test showed that under intense light and high temperatures, the PDI increased but was still <0.3 within 30 days, compared to 0 days (0.136). The results showed that the stability of osthol in PBCA-NPs was less affected by high light and high temperatures than by free osthol. During storage, the size of the deposited particles increased due to the deposition phenomenon, while the zeta potential decreased due to the absorption of Poloxamer-127 on the surfaces of the NPs. However, these changes were within a reasonable range and had no significant effect on the quality of the osthol-PBCA NPs.

### 3.4. In Vitro Drug Release Study

**Figure 5:**
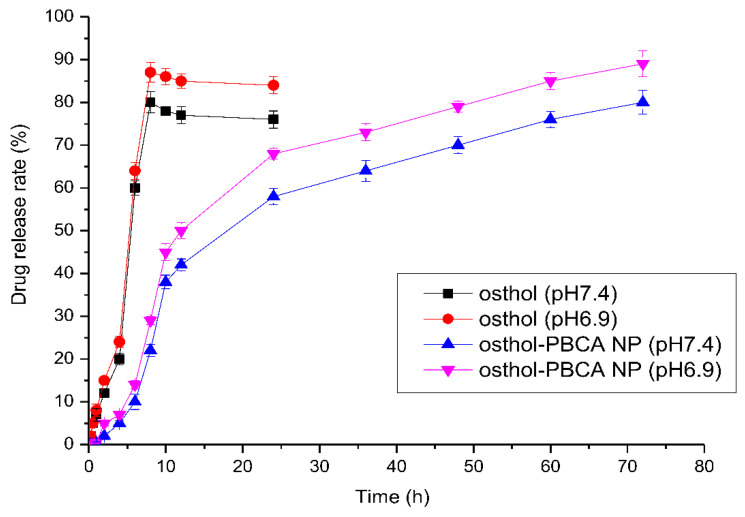
The drug release process of osthol and osthol-PBCA NPs in vitro.

The release of osthol from osthol-PBCA NPs was studied in a dynamic dialysis bag in vitro. The drug–time release curve is shown in Figure 5. Obviously, in PBS solution at pH 7.4, 80% of osthol was released from the osthol solution within 8 h, while the drug release from the NPs was slow and less than 60% of osthol was released from the osthol-PBCA NPs in 36 h, and 80% release occurred after 72 h. Controlled/slow release occurred, with 10% of the osthol being released from the nanoformulation every seven hours. In PBS solution at pH 6.9, the drug release rate at the corresponding time increased by 10%, indicating that the drug release system was still in a slow-release mode in the tumor environment. The drug release rate of drug-loaded PBCA NPs is mainly affected by the degradation rate, and the factors affecting the degradation of PBCA NPs may include pH, which needs to be further researched. This type of delivery system will significantly reduce the dosing frequency in the therapeutic process, improve patients’ compliance, reduce the blood drug concentration fluctuations in vivo, and maintain blood drug concentrations within the therapeutic range, along with reduced drug side effects. 

### 3.5. Kinetics of Intracellular Endocytosis of Osthol-PBCA NPs

**Figure 6:**
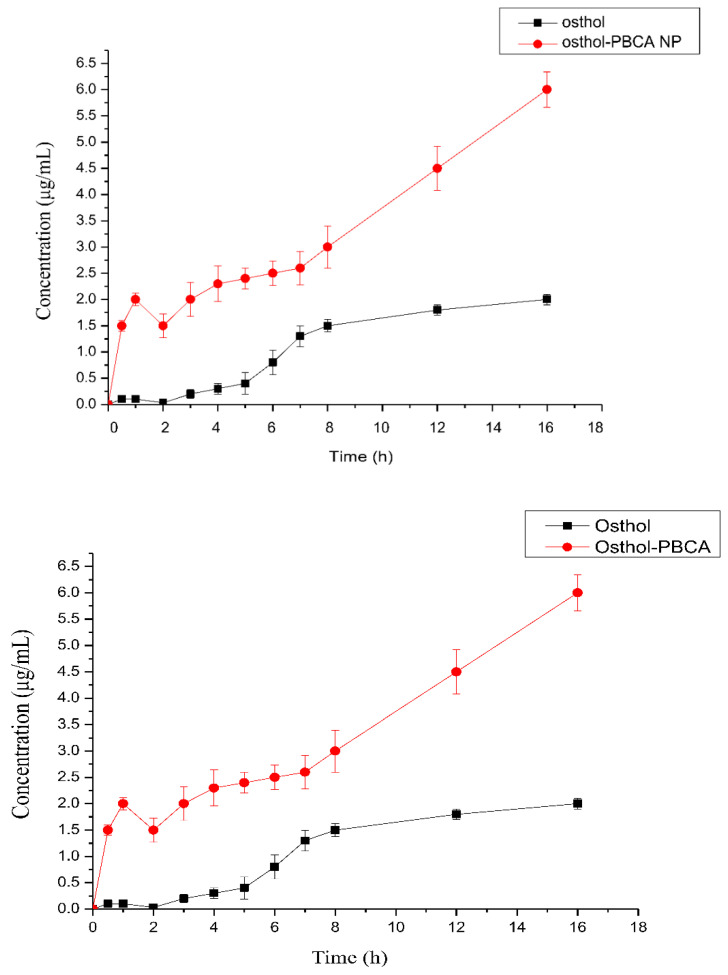
Process of intracellular endocytosis of osthol-PBCA NPs in SH-SY5Y cells.

To determine the uptake process of the osthol solution and osthol-PBCA NPs into SH-SY5Y cells, the absorption of osthol was determined at different time points. The results revealed that the concentration of osthol increased as the incubation time increased (Figure 6). The uptake of osthol-PBCA NPs was also more efficient than the uptake using osthol serum-free medium solution (10 μg·mL^−1^) at 0.5, 1, 2, 3, 4, 5, 6, 7, 8, 12, and 16 h. The curve showing the kinetics of absorbed amounts over the incubation time (1–16 h) is shown. The number of nanoparticles entering the cell reached a plateau after 8 h, indicating that the number of nanoparticles transported into cells had reached saturation. According to the first-order reaction kinetics, the rate constant was determined as k = (0.2754 ± 0.046) h^−1^. The uptake of osthol in SH-SY5Y cells by osthol-PBCA NPs was three-fold faster than for free osthol. 

### 3.6. The Endocytosis Transport Mechanism of Osthol-PBCA NPs in Cells

**Figure 7:**
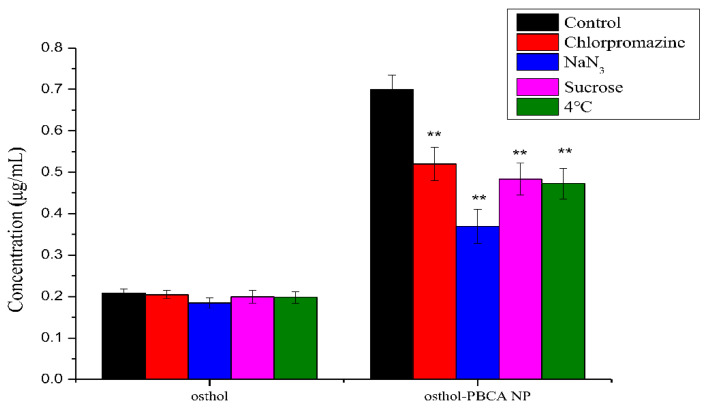
The uptake process of osthol and osthol-PBCA NPs in SH-SY5Y cells using endocytosis inhibitors or at a low temperature (** *p* < 0.01 vs. osthol group).

Endocytosis mechanisms show how cells interact with the environment. Different types of drug endocytosis mechanisms lead to different drug uptake rates. The main mechanisms of endocytosis are clathrin-mediated endocytosis and receptor-independent macropinocytosis. NPs different in size, charge, shape, and core material regulate different endocytosis processes, and the cell adjusts these pathways to meet its physiological needs. In this study, the cellular uptake mechanism of osthol solution and osthol-PBCA NPs by SH-SY5Y cells was studied using endocytic inhibitors (Figure 7). 

The SH-SY5Y cell temperature had a greater effect on the osthol-PBCA NP absorption than the osthol solutions themselves. When the culture temperature was reduced to 4 °C, the uptake of osthol-PBCA NPs was decreased by 32% (Figure 7). Low-temperature test results show that osthol-PBCA NPs appear to be uptaken by SH-SY5Y cells via an energy-dependent and active transport process, whereas free osthol entered the cell primarily through other routes that do not require energy, such as passive diffusion. In addition, the fluidity of cell membrane lipids will be reduced at a low temperature (0 °C), which will affect the interaction between exogenous substances and cell membranes, leading to a low uptake rate of exogenous substances [35].

As shown in Figure 7, the absorption of free osthol was not significantly affected by the inhibitors. In contrast, the uptake of osthol-PBCA NPs by cells was significantly reduced by the inhibitors. NaN_3_ can block the formation of intracellular ATP by inhibiting cytochrome oxidase to block mitochondrial exhalation chains. When treated with NaN_3_, the uptake of osthol-PBCA NPs into cells was inhibited by 45.7%, indicating that the endocytosis of nanoparticles is an active, energy-dependent process. Sucrose disrupts the formation of clathrin-coated vesicles to impact the clathrin-mediated endocytosis pathway. When treated with sucrose, the uptake of nanoparticles was inhibited by 32%, indicating that the clathrin-mediated endocytosis pathway exists. Chlorpromazine is a clathrin-mediated endocytic pathway-specific inhibitor; it lowered the inhibition rate by 24%. This level of inhibition serves as an indicator that nanoparticles entered cells through the clathrin-mediated endocytosis pathway. 

These cellular uptake results imply that the intracellular endocytosis of osthol-PBCA NPs is an energy-, time-, and clathrin-dependent process and involves all, but to varying degrees, of the investigated cellular uptake mechanisms. This result is consistent with clathrin-mediated endocytosis (CME) being the predominant mode of uptake in eukaryotic cells [36]. Other mechanisms of drug absorption may also exist and require further investigation.

### 3.7. In Vitro Cytotoxicity Studies

**Figure 8:**
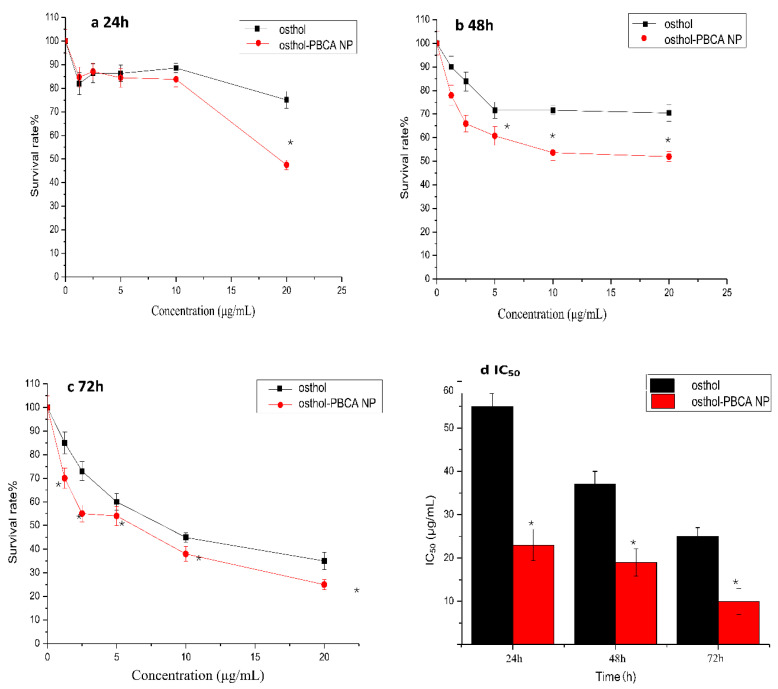
The survival rate and IC_50_ of SH-SY5Y cells incubated with osthol and osthol-PBCA NPs for 24, 48, and 72 h ($\bar{Χ}$ ± S, *n* = 4, * *p* < 0.05 vs. control).

In the present study, PBCA NPs without osthol were prepared parallelly and served as the negative controls, which showed non-toxicity to SH-SY5Y cells (data no show). Results of in vitro cytotoxicity studies are shown in Figure 8; the data showed that the cytotoxicity of osthol was significantly increased after the preparation of osthol-PBCA NPs (*p* < 0.05) in SH-SY5Y cell lines. When the drug concentration was higher than 5 μg·mL^−1^ and the incubation time was longer than 24 h, the cytotoxicity of osthol-PBCA NPs was significantly higher than that of osthol (*p* < 0.05) and increased by approximately 10–20%, and it was more prominent at 48 h and 72 h.

IC_50_ evaluation could be useful for the better characterization of the anticancer performance of the proposed drug delivery system. The IC_50_ of osthol-PBCA NPs (23 μg·mL^−1^) was significantly (*p* < 0.05) lower than in the osthol group (55 μg·mL^−1^) in the 24 h evaluation. The IC_50_ value decreased with increasing incubation time. The cytotoxicity of osthol-PBCA NPs relative to osthol may originate from the sustained drug release from the DL NPs. It may also be related to the fact that more NPs enter the cells much faster via the endocytosis function. The results showed that the proposed drug delivery system has promising anticancer performance. This result is consistent with reports that the encapsulation of drugs by nanocarriers can improve the toxicity of drugs to tumor cells [37,38].

### 3.8. Osthol-Induced LDH Determination

Compared with control SH-SY5Y cells (0.03), the levels of LDH in the cellular supernatant of SH-SY5Y cells treated with 10 μg·mL^−1^ osthol and osthol-NPs (0.18, 0.34) were significantly increased. These results indicated that osthol-NPs (10 mmol·L^−1^) promoted LDH release in SH-SY5Y cells. Compared with the osthol group, the level of LDH in the osthol-NP treatment group was decreased, suggesting that osthol-NPs significantly enhanced the cytotoxicity of osthol (10 μg·mL^−1^) to SH-SY5Y cells within 24 h (*p* < 0.05).

### 3.9. The PK and AUC Study in Rats

**Figure 9:**
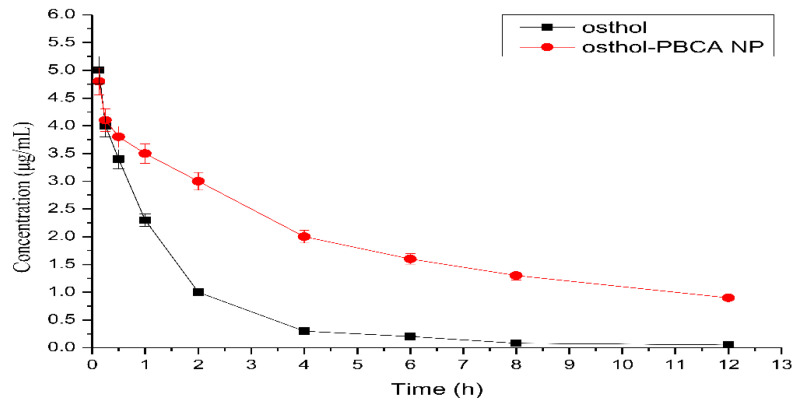
Plasma drug concentration–time cures of osthol after administration of 5 mg·mL^−1^ osthol free or in osthol-PBCA NP suspension via the tail veins of rats.

The changes in time–plasma concentrations after the intravenous administration of 5 mg/mL osthol and osthol-NP suspensions in rats are shown in Figure 9 and the PK parameters calculated using 3P87 are shown in Table 7. The PK characteristics of both free osthol and osthol in PBCA-NPs both fit the two-compartment model.

The encapsulation of osthol in the PBCA-NPs caused a considerable change in the PK parameters. The results showed that the AUC of osthol-NPs (22.52 μg/mL∙h) was 3.3-fold higher than that of free osthol (6.90 μg/mL∙h); the CL of osthol-PBCA NPs was 0.278 ± 0.021 (mg)/(μg/mL)/h, which was 6.8-fold lower than that of the osthol suspension (*p* < 0.05). The improved bioavailability might be ascribed to the cells’ endocytosis mechanism of NPs, which should be investigated in detail. The results indicate that PBCA-NPs can be a promising carrier for the construction of an i.v. controlled-release delivery system with osthol. 

## 4. Conclusions

NB is considered one of the most aggressive human cancers; high-risk NB is potentially lethal. Osthol is a promising agent for the treatment of cancer. However, the low water solubility of osthol limits its potential clinical application. Therefore, to improve the aqueous solubility of the drug, osthol was incorporated into PBCA NPs by a microemulsion polymerization technique. The obtained osthol-PBCA NPs were spherical, with good dispersion, a small particle size, and a good PDI; the drugs in osthol-NPs had better stability and sustained release mode activity. The osthol-loaded nanoparticles displayed significantly enhanced intracellular uptake and cytotoxicity compared with free osthol; in vivo pharmacokinetic studies revealed that the AUC of osthol-NPs was higher than that of free osthol. The toxicity of osthol to the cell membrane was increased after nanosizing, and the rate and amount of absorption were increased. These findings also suggest that osthol-PBCA NPs can be used as a viable and bioavailable alternative to improve their bioavailability and have potential efficacy in the treatment of NB. 

## Figures and Tables

**Table 1:** Variables and levels in the orthogonal design. Osthol dosage was 5 mg; stirring speed (A), pH value (B), α-BCA (C), and Poloxamer-127 (D) were chosen as the research factors, with 3 levels for each factor.

	Variable	A/Speed (r/min)	B/pH	C/α-BCA (μL)	D/Poloxamer-127 (mg)
Levels					
1		800	2	10	10
2		1000	4	15	20
3		1200	6	20	40

**Table 2:** Single-factor investigation: particle size.

Speed (r/min)	Average Size (nm)	PDI	Maximum Size (nm)	Size Distribution
800	163.4	0.148	201.3	102.2
1000	137.3	0.135	153.8	62.13
1200	101.8	0.125	113.0	35.32

**Table 3:** Results of the orthogonal test. The orthogonal experimental design table L^9^ (34) was used to optimize the preparation, and the most suitable were screened for the encapsulation rate as shown by the index.

	Variable	A (r/min)	B (pH)	C (α-BCA μL)	D (mg)	Encapsulation Rate%
Levels						
1		800	2	10	10	70.53
2		800	4	15	20	73.99
3		800	6	20	40	72.45
4		1000	2	15	40	79.08
5		1000	4	20	10	77.52
6		1000	6	10	20	77.19
7		1200	2	20	20	77.43
8		1200	4	10	40	77.85
9		1200	6	15	10	78.81
K1		72.312	75.679	75.189	75.621	
K2		77.932	76.452	77.296	76.203	
K3		78.028	76.142	75.787	76.449	
R		5.716	0.773	2.107	0.828	

K1–K3 refers to the importance of level 1–3 of variable to the EE%. R refers the extreme value, R = K_max_−K_min_.

**Table 4:** Analysis of variance of orthogonal experimental data.

Factor	SS	DOF	F Ratio	Critical Value	Significance
A (r/min)	64.242	2	16.098	6.94	<0.05
B (pH)	0.907	2	0.227	6.94	>0.05
C (α-BCA μL)	7.077	2	1.773	6.94	>0.05
D (mg)	1.085	2	0.272	6.94	>0.05
Error	7.98	4			

**Table 5:** Particle size, zeta potential, and EE of the optimum osthol-PBCA NPs (*n* = 3).

Formulations	Size (nm)	Size Distribution	PDI	Zeta Potential (mV)	Encapsulation Efficiency (%)	Loading Efficiency (%)
Osthol-loaded PBCA nanoparticles	110 ± 6.7 nm	20.3 nm	0.126 ± 0.014	−13 ± 0.32 mV	80.59	40%
PBCA nanoparticles	96 ± 5.3 nm	18.3 nm	0.123 ± 0.013	−7 ± 0.40 mV	−	−

**Table 6:** Influencing factors test of osthol-PBCA-NP suspensions and freeze-dried powder over a period of 30 days (*n* = 10).

(**a**) Optical Stability Test (3000 lux)							
	Time (day)	0	2	7	14	30	Degradation Rate (%)
Content (%)							
Osthol		100 ± 5.70	86 ± 6.70	60 ± 4.50	43 ± 5.50	32 ± 6.80	68
Osthol-PBCA-NP suspensions		100 ± 6.10	94 ± 6.80	90 ± 5.41	88 ± 6.40	86 ± 5.65	14
Osthol-PBCA-NP powder		100 ± 5.30	99 ± 4.60	90 ± 5.40	98 ± 3.12	94 ± 5.60	6
(**b**) Thermal stability test (60 °C)							
	**Time (day)**	**0**	**2**	**7**	**14**	**30**	**Degradation Rate (%)**
**Content** **(** **%** **)**							
Osthol		100 ± 5.40	76 ± 6.50	52 ± 3.50	35 ± 5.50	28 ± 6.80	72
Osthol-PBCA-NP suspensions		100 ± 5.30	93 ± 6.40	89 ± 5.40	87 ± 6.40	84 ± 5.65	16
Osthol-PBCA-NP powder		100 ± 4.70	99 ± 3.60	97 ± 5.80	94 ± 3.52	90 ± 4.60	10

**Table 7:** PK parameters of osthol and osthol-PBCA NPs in rats ($\bar{Χ}$ ± S, *n* = 9).

Parameters	Unit	Osthol	Osthol-PBCA NP
t_1/2 α_	min	0.328 ± 0.235	0.269 ± 2:035
t_1/2 β_	min	28.135 ± 5.65	230.8 ± 7.54
AUC	μg/mL∙h	6.90 ± 1.831	22.52 ± 5.76
CL	(μg∙h /mL)	1.89 ± 0.4	0.278 ± 0.021

## Data Availability

Data is contained within the article.
